# Low Temperature Enhances N‐Metabolism in *Paxillus involutus* Mycelia In Vitro: Evidence From an Untargeted Metabolomic Study

**DOI:** 10.1111/1462-2920.70162

**Published:** 2025-08-12

**Authors:** Agnieszka Szuba, Weronika B. Żukowska, Joanna Mucha, Aleksander Strugała, Łukasz Marczak

**Affiliations:** ^1^ Polish Academy of Sciences Institute of Dendrology Kórnik Poland; ^2^ Faculty of Forestry and Wood Technology Poznań University of Life Sciences Poznań Poland; ^3^ Polish Academy of Sciences Institute of Bioorganic Chemistry Poznań Poland

**Keywords:** carbohydrates, decreased growth, ectomycorrhizal fungi, high‐throughput GC MS/MS, low temperature exposure, metabolomics, N‐compounds

## Abstract

This metabolomic study investigates, using GC MS/MS analysis, the molecular response of *Paxillus involutus* mycelia to prolonged low temperature (4°C) exposure. Alongside reduced growth, decreased overall nutrient levels, and increased oxidative stress indicators, analyses revealed a significant increase in nitrogen (N) concentration and enhanced N metabolism, particularly via the GS–GOGAT pathway, which was associated with elevated concentrations of numerous amino acids. In contrast, carbon (C) metabolism was not intensified but largely reprogrammed, with varying changes in carbohydrate abundance but higher levels of several stress‐related metabolites, such as trehalose and inositol family members, indicating activation of tolerance mechanisms, all with unchanged C (%). These changes suggest enhanced NH_4_^+^ uptake and a redirection of glycolysis‐derived C skeletons towards N‐compound biosynthesis. The lack of massive upregulation of typical anti‐stress compounds under low temperature exposure indicates either acclimatisation or mild stress. Mycelial restructuring, including increased dry mass (%) and accumulation of chitin precursors, implies cell wall remodelling and cold acclimatisation, supported by changes in membrane components. All these findings suggest that low temperatures may enhance N metabolism in ECM fungi even without additional carbon supply, potentially affecting symbiotic balance under climate change. Further studies are needed to validate these mechanisms and ecological implications.

## Graphical abstract

Under low‐temperature exposure, *Paxillus involutus* mycelium cultivated in vitro exhibited strong growth inhibition and mild stress response. At the same time, an increased abundance of numerous N‐compounds and metabolites involved in providing C‐skeletons for enhanced AA biosynthesis was detected in mycelial cells (as shown by high‐throughput GC–MS/MS analysis).

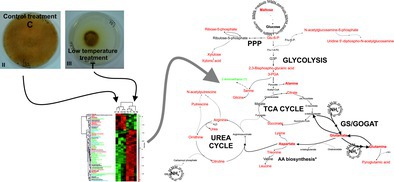

**Abbreviations**
Arg: arginine
ECM: ectomycorrhiza
ECMF: ectomycorrhizal fungi
GC MS/MS: gas chromatography tandem mass spectrometry
GlnNAc: N‐acelylglucosamine
Gly: glycine
GS—GOGAT: the glutamine synthetase—glutamate synthase pathway
MDA: malondialdehyde
MMN: modified Melin–Norkrans agar medium
ROS: reactive oxygen species

## 1 Introduction

As major symbionts of vascular plants, ectomycorrhizal fungi (ECMF) significantly influence nutrient and water uptake, stress resistance, growth, and other aspects of tree functioning (Kottke et al. 1987; Martin et al. 1994; Treseder 2013; Lazarević et al. 2016; Ma, He, et al. 2014; Bogar et al. 2022). *Paxillus* is one of the most important and frequently analysed ECMF genera with a broad distribution across the northern hemisphere (Li et al. 2020; Usman et al. 2021). Its residual saprotrophic capabilities (Doré et al. 2015; Heinonsalo et al. 2015) make it easy to establish new in vitro lines and cultivate them in laboratory conditions (Langenfeld‐Heyser et al. 2007; Muller et al. 2013) as well as to inoculate vascular plants (Muller et al. 2013; Gafur et al. 2004; Li et al. 2012; Ma, Sun, et al. 2014; Szuba, Marczak, and Ratajczak 2020). Researchers often use in vitro experimental models, although they are artificial and deprived of many contextual factors existing in natural environments (Poorter et al. 2012). Controlled conditions make it possible to investigate the molecular background of biological processes and to determine the factors influencing molecular shifts (e.g., the effect of the presence of the symbiotic partner during an ectomycorrhizal association) (Muller et al. 2013; Gafur et al. 2004; Szuba, Marczak, Ratajczak, Kasprowicz‐Maluśki, et al. 2020). The majority of molecular, including metabolomic, studies on ectomycorrhizal fungi‐plant symbioses focus, however, on the plant molecular response to the presence of the fungus (Gafur et al. 2004; Szuba, Marczak, Ratajczak, Kasprowicz‐Maluśki, et al. 2020; Tschaplinski et al. 2014; Vayssieres et al. 2015), whereas the molecular status of the mycelium itself is often overlooked.

The majority of biochemical and omic research on ECM has been carried out under ‘classical’ laboratory conditions (about 21°C) (Ma, He, et al. 2014; Heinonsalo et al. 2015; Muller et al. 2013; Gafur et al. 2004; Li et al. 2012; Ma, Sun, et al. 2014; Szuba, Marczak, and Ratajczak 2020; Szuba, Marczak, Ratajczak, et al. 2020; Tschaplinski et al. 2014). Based on laboratory studies, the thermal optimum of ECMF, judged by the maximum radial growth of the mycelium (Jeon et al. 2012), has been established between 20°C and 30°C (Pietikainen et al. 2005; Lazarevic et al. 2016). Moreover, thanks, among other things, to research on the impact of climate change we know more about the fungal response to soil warming (than cooling) (Usman et al. 2021; Rudawska and Leski 2019; Treseder et al. 2016; Ma et al. 2018). Higher temperatures have for example a positive effect on ectomycorrhizal taxa that are specialists for conditions with high N availability and responded positively to inorganic N additions (Lilleskov et al. 2011; Solly et al. 2017) as higher temperature increases the availability of inorganic N pool (Solly et al. 2017). Unlike reports on studies conducted at laboratory temperatures or on soil warming, molecular research on the impact of low temperature on ECMF is sparse (Usman et al. 2021; Liu et al. 2018). At the same time, ECMFs are predominantly occurred in the habitats of the northern hemisphere characterised with the relatively cold climate (Li et al. 2020; Usman et al. 2021; Tibbett and Cairney 2007), thus are frequently exposed to low temperatures under natural conditions. Fungi are generally considered to be better adapted to lower temperatures than other soil organisms (Pietikainen et al. 2005). Furthermore, ectomycorrhizal fungi may grow even in sub‐zero temperatures with lethal temperatures identified for selected ECMFs as low as −10°C (Marx et al. 1970; Lehto et al. 2008). Therefore, more thorough knowledge about ECMF metabolism at low temperatures seems urgently needed. The temperature used in our study (4°C) is typical not only for mycelia long‐term laboratory storage (Tibbett et al. 1999), but also for natural conditions. This allowed us to highlight the metabolomic changes that can occur in ECMF mycelium growing in nature, for example when it is exposed to temperatures common in temperate forests during spring and autumn.

Although the response of ectomycorrhizal pure cultures to low temperatures has not yet been studied at the metabolomic level, we can draw some information from experiments on co‐called psychrophilic fungi—genera adapted to even lower temperatures than applied in our study (Solly et al. 2017; Tibbett and Cairney 2007; Tibbett, Sanders, Minto, et al. 1998; Su et al. 2016). It was shown, for example, that under stress conditions psychrophilic ectomycorrhiza genotypes biosynthesize and exude more cold‐active enzymes involved inter alia in nutrient acquisition (Tibbett, Sanders, Minto, et al. 1998; Tibbett, Sanders, and Cairney 1998; Abu Bakar et al. 2020). Other works have suggested that the ability to utilise free amino acids is a key ecological adaptation to low temperature in cold‐adapted fungal ecotypes (Tibbett and Cairney 2007; Tibbett, Sanders, Minto, et al. 1998). Those results suggest an important role of primary N‐metabolism in response to low temperature. To verify this hypothesis, we used a non‐targeted high‐throughput Gas Chromatography—Tandem Mass Spectrometry (GC MS/MS) approach to examine the primary metabolites, such as carbohydrates as well as amino acids and other N‐compounds or intermediates of the core metabolic cycles, in vitro growing *P. involutus* exposed long‐term to 4°C.

Conditions used in our study are commonly regarded as low temperature stress (Zhu et al. 2017). Previous studies suggested that fungi respond to low temperature by modifying their energy metabolic pathways, usually decreasing their activity (Abu Bakar et al. 2020; Atkin and Macherel 2009; Liu et al. 2015). The tolerance (and adaptability) mechanisms involved increased biosynthesis of trehalose, osmoprotectants (like mannitol and glycerol), and pigments or cell wall melanisation (Lehto et al. 2008; Su et al. 2016; Hassan et al. 2016). Increased abundance of various osmoprotectants will enhance the tolerance of mycelium to low temperatures also by protecting membranes from negative effects of intracellular mycelia dehydration (Lehto et al. 2008). Abiotic stress, including a low temperature one, usually caused a strong radical burst resulting in macromolecule damage like peroxidation of membrane lipids (Zhu et al. 2017; Kostadinova et al. 2012) or protein degradation (Abu Bakar et al. 2020; Kostadinova et al. 2012). At the same time, the lipid composition has changed (an increase in the proportion of polyunsaturated or branched lipids) to maintain membrane fluidity and prevent electrolyte leakage (Kostadinova et al. 2012). Chaperone biosynthesis was increased to prevent protein degradation (Abu Bakar et al. 2020). The common effect of abiotic stress is, therefore, the redirection of cell metabolism into the biosynthesis of defence/protective compounds, which consequently results in growth inhibition (Szuba, Marczak, Ratajczak, Kasprowicz‐Maluśki, et al. 2020; Ramakrishna and Ravishankar 2011). For these reasons, except for the alterations in primary metabolism, we expected to observe other common symptoms of low temperature stress response in *P. involutus* mycelia. To address this hypothesis, we analysed common markers of oxidative stress (i.e., levels of ROS and marker of cell membrane integrity) commonly observed in response to various abiotic stresses (Ma, He, et al. 2014). The verification of stress levels in *Paxillus* mycelium exposed to low temperature seems particularly important in the context of the postulated ECMFs adaptation to the cold conditions of the northern hemisphere (Li et al. 2020; Usman et al. 2021; Tibbett and Cairney 2007).

Our goal was to provide new information on molecular pathway shifts in *P. involutus* under low temperature exposure. Studying the response of ECMF to low temperature can help us to understand fungal responses to temperature changes in general, aiding predictions for future climate scenarios (which would include both increases and decreases to local temperatures) and to better inform forest management and conservation practices.

## 2 Material and Methods

### 2.1 Fungal Culture and Treatment

The *Paxillus involutus* strain, obtained from a fruit body growing in a poplar monoculture, was barcoded, introduced into an in vitro culture, and maintained in Petri dishes in a Modified Melin–Norkrans agar medium (MMN; (Kottke et al. 1987)); for the exact composition of the grown medium (see: Appendix A). The *P. involutus* genotype analysed in this study was previously genotyped and tested in our lab in the context of ectomycorrhizal interaction with *Populus* × *canescens* (Szuba, Marczak, and Ratajczak 2020; Szuba et al. 2016). In the present experiment, 5 × 5 mm fragments of fresh mycelia were transferred to Petri dishes with an agar medium covered with cellophane. Mycelia grew in two independent growth chambers either in controlled conditions (in darkness at 21°C ± 1°C; treatment ‘C’), or exposed to low temperature—mycelia kept in darkness at 4°C ± 1°C throughout the whole experiment (treatment ‘T’).

### 2.2 Biometrical Analysis

The diameter of mycelia was measured from high‐resolution images captured at 2, 4, and 6 weeks of growth using ImageJ v1.48 software (Wayne Rasband, Bethesda, MD, USA) (*n* = 30 for controls and 34 for low temperature exposed mycelia). Additionally, the pigmentation of the agar growth media was estimated by measuring the maximum grey values of images of agar taken at the end of the experiment and next converted into the grey scale (using ImageJ v1.48 software). The high‐resolution images of mycelia were also used to document phenotypic changes in the mycelium.

After 6 weeks of growth, before the entire surface of the Petri dishes was overgrown by the fastest growing individuals (to avoid nutrients/carbon limitation), the mycelia were harvested. The number of sclerotial bodies and the fresh mycelial mass (devoid of sclerotial bodies) were measured. Samples of mycelia intended for biochemical and metabolomic studies were collected (about 20–50 mg of fresh mycelia from each plate), immediately frozen in liquid nitrogen, and stored at −80°C until analysed. Five to 10 randomly selected mycelia frozen fragments (each represented mycelium collected from individual plate) were pooled and homogenised under liquid nitrogen to create each particular sample used subsequently for biochemical and metabolomic analysis.

The remaining mycelia were harvested and dried at 60°C for 48 h. Dry mass was used for further analysis (e.g., elemental analysis). Mycelia dry mass percentage was determined according to the formula DM % = (DW/FW) × 100%. DM % was used for example, to estimate the whole mycelia dry mass.

Representative sclerotial bodies from control treatment (*n* = 3) were fixed, cut into 20 μm cross‐sections, stained with 0.05% toluidine blue O (Sigma, St Louis, MO, USA), and then analysed microscopically using an Axioscope 20 microscope (Carl Zeiss, Jena, Germany; equipped with a 40× lens) as described previously (Szuba, Marczak, Ratajczak, Kasprowicz‐Maluśki, et al. 2020).

### 2.3 Mineral Analysis

Samples of dried mycelia (devoid of sclerotial bodies; 10 mg, *n* = 12) were ground into a powder in a ball mill, mineralised and analysed to determine their concentrations of Mg, K, Ca, Na, Fe, Co, Cu, and Zn. The samples were analysed in a microwave mineraliser (Multiwave 3000, Anton Paar, GmbH, Austria) and an inductively coupled plasma time‐of‐flight (TOF) mass spectrometer (Spectrometer OptiMass 9500 ICP‐TOF‐MS, GBC Scientific Equipment, Hampshire, Braeside, Australia) owned by the Institute of Dendrology, Polish Academy of Sciences, Kórnik, Poland. For all runs, we used certified reference material (NCS DC 73349 Bush Branches and Leaves; China National Analysis Center for Iron & Steel, Beijing, China). Total nitrogen and carbon were determined in dried and ground mycelia (*n* = 3) with a CHNS analyser (2400 CHNS/O Series II System, PerkinElmer, Waltham, MA, USA), after which the C/N ratio was calculated. Data normalised with fresh and dry mass are presented in Table 1.

**TABLE 1:** Concentrations of elements in *Paxillus involutus* mycelia normalised by both, dry and fresh mass.

Element	Normalised by DW				Normalised by FW			
	C	Trend^a^	T	*p*	C	Trend^a^	T	*p*
C (%)	44.17 ± 0.28	↔	44.27 ± 0.09	0.750	5.03 0.03 ±	↑	6.15 ± 0.01	< 0.001
N (%)	2.16 ± 0.03	↑	3.09 ± 0.13	< 0.001	0.246 ± 0.004	↑	0.428 ± 0.010	< 0.001
C/N	23.81 ± 0.22	↓	16.74 ± 0.40	< 0.001	—			
Mg (μg g ^−1^)	2243 ± 23	↓	926 ± 31	< 0.001	255.1 ± 2.6	↓	128.3 ± 4.3	< 0.001
P (μg g ^−1^)	11,973 ± 196	↓	6649 ± 253	< 0.001	1362 ± 22	↓	921 ± 35	< 0.001
K (μg g ^−1^)	21,013 ± 352	↓	15,310 ± 151	< 0.001	2391 40	↓	2122 72	0.036
Ca (μg g ^−1^)	799 ± 34	↓	543 ± 103	0.028	90.89 3.9	↔	75.32 14.3	0.305
Na (μg g ^−1^)	210.6 ± 6.0	↓	143.3 ± 6.7	< 0.001	23.96 0.68	↓	19.86 0.93	0.002
Fe (μg g ^−1^)	630.7 ± 26.1	↓	250.6 ± 8.6	< 0.001	71.75 3.0	↓	34.72 1.2	< 0.001
Cu (μg g ^−1^)	20.02 ± 0.26	↓	13.19 ± 0.52	< 0.001	2.28 0.03	↓	1.83 0.07	< 0.001
Zn (μg g ^−1^)	55.73 ± 14.17	↓	23.58 ± 0.68	0.034	6.34 1.6	↔	3.27 0.09	0.070

*Note:* Values measured with the use of dry mass, concentrations in fresh weight calculated according to average dry mass (%). Treatments: C‐control; T‐low temperature stress. ↓‐less in the treated mycelium; ↑‐more in the treated mycelium, ↔‐no significant differences in element concentration. The results are presented as the mean value ± standard error for 3 independent replicates for C and N, and for 12 independent replicates for the remained elements. Significant differences between treatments are calculated according to the *T* test (*α* = 0.05).

^a^ Trend between T and C.

### 2.4 MDA and H_2_O_2_

H_2_O_2_ content was tested according to Zhou et al. (2006). 250 mg of the frozen fungal hyphae powder (*n* = 6; for sample composition: see Section 2.1) was suspended at 4°C in activated carbon and 5% trichloroacetic acid (TCA) at the ratio of 1: 0.2: 1 (w/w/v). H_2_O_2_ concentration was determined using a colorimetric reagent made by mixing 0.6 mM 4‐ (2‐pyridiazole) resorcinol disodium salt with 0.6 mM titanium potassium oxalate at a 1:1 ratio in 50 mM phosphate buffer (pH 8.4); absorption was measured at λ = 508 nm. The concentration of hydrogen peroxide was read from a standard curve and was reported as μmol per g fresh mycelium (μmol g‐1 FW). Additionally, data were normalised with dry weight.

To estimate the intensity of lipid peroxidation in *P. involutus* hyphae, we determined malondialdehyde (MDA) concentration in frozen mycelia homogenised in 0.25 M H_2_SO_4_/10% TCA solution according to modified methods of Shah et al. (Shah et al. 2001). Thiobarbituric acid (TBA) was added to the extract mixture (to make 0.25% of the mixture's vol.). After 10 min of boiling, the reaction of TBA with MDA was stopped by cooling (on ice). The supernatants were analysed at 532 nm to determine the reaction products and at 600 nm for nonspecific reaction products, calculated per g FW (Stobrawa and Lorenc‐Plucińska 2008) as well as normalised with dry weight.

### 2.5 Metabolome Study

Metabolites for GC–MS/MS were isolated from 20 mg samples of mycelia (*n* = 4; for sample composition: see Section 2.1) by methanol extraction and were further derivatized using a standard procedure, as reported previously (Szuba, Marczak, Ratajczak, Kasprowicz‐Maluśki, et al. 2020). The extracts were analysed with an Agilent 7890A gas chromatograph coupled to a Pegasus 4D GCxGC‐TOFMS mass spectrometer (LECO, St. Joseph, MI, USA). The compounds were separated using a DB‐5 T bonded‐phase fused‐silica capillary column (30 m length, 0.25 mm inner diameter, 0.25 μm film thickness) (J&W Scientific Co., USA). The GC oven temperature programme was as follows: 2 min at 70°C, raised by 10°C/min to 300°C and held for 10 min at 300°C. The GC analysis lasted a total of 36 min. Helium was used as the carrier gas at a flow rate of 1 mL/min. One microliter of each sample was injected in splitless mode. The initial injector temperature was 40°C for 0.1 min, after which the temperature was raised by 6°C/min to 350°C. The septum purge flow rate was 3 mL/min and the purge was turned on after 60 s. The transfer line and ion source temperatures were set to 250°C. In‐source fragmentation was performed at 70 eV. Mass spectra were recorded in the range between 35 and 850 m/z. Data acquisition, automatic peak detection, mass spectrum deconvolution, retention index calculation and library searches were performed using LECO ChromaTOF software. To eliminate retention time (Rt) shift and determine retention indexes (RI) for each compound, an alkane series mixture (C‐10 to C‐36) was injected into the GC/MS system. The metabolites were identified automatically with a library search (NIST library); an analyte was considered identified when it passed a quality threshold, that is, a similarity index (SI) above 700 and a matching retention index ±10. Artefacts (alkanes, column bleed, plasticizers, MSTFA and reagents) were identified using the same method, and then excluded from further analyses. To obtain accurate peak areas for the deconvoluted components, unique quantification masses for each component were specified and the samples were reprocessed. The obtained profiles were normalised against the sum of the chromatographic peak areas (using the TIC approach).

For duplicate identifications of the same metabolite, its ion intensities were summed (Appendix C). The resulting data tables were used for statistical analysis using Perseus 1.6.15.0 software (Max Planck, Martinsried, Germany). Additionally, a quantitative enrichment analysis (using intensities of all identified compounds) and an enriched metabolite sets analysis (using the list of compounds significantly upregulated under a particular treatment, according to t‐tests) were performed using the MetaboAnalyst 6.0 (https://www.metaboanalyst.ca/) platform with default settings, using the KEGG database.

### 2.6 Statistics

Biometrical and biochemical mycelia results were analysed using JMP Pro 13.0.0 software (SAS Institute Inc.). Values were considered significant at *p* < 0.05 as determined with *t*‐tests. The data tables used for the metabolome study were analysed using Perseus ver. 1.6.15.0 software (Max Planck) and MetaboAnalist 6.0 platform. The ion intensities were transformed to log values and all samples were grouped using a categorical annotation. The values were filtered for blanks in samples (min. 75% presence in at least one group; *n* = 4). The missing values in Perseus' data table were replaced from the normal distribution, and the prepared matrices were used for statistical calculations in Perseus and MetaboAnalist. PCA and Volcano plot analyses were carried out on the data. False discovery rate (FDR) statistical measure was used for multiple testing of the metabolome data to avoid type I error. Clustering analysis was performed on data normalised using the *Z*‐score algorithm. Only identified metabolites that had significantly different abundances (according to the t‐test (α = 0.05); FDR = 0.05, number of randomizations = 250) were presented on the heat map.

## 3 Results

### 3.1 Phenotypic and Biometric Changes in Mycelia Exposed to Low Temperature

**FIGURE 1:**
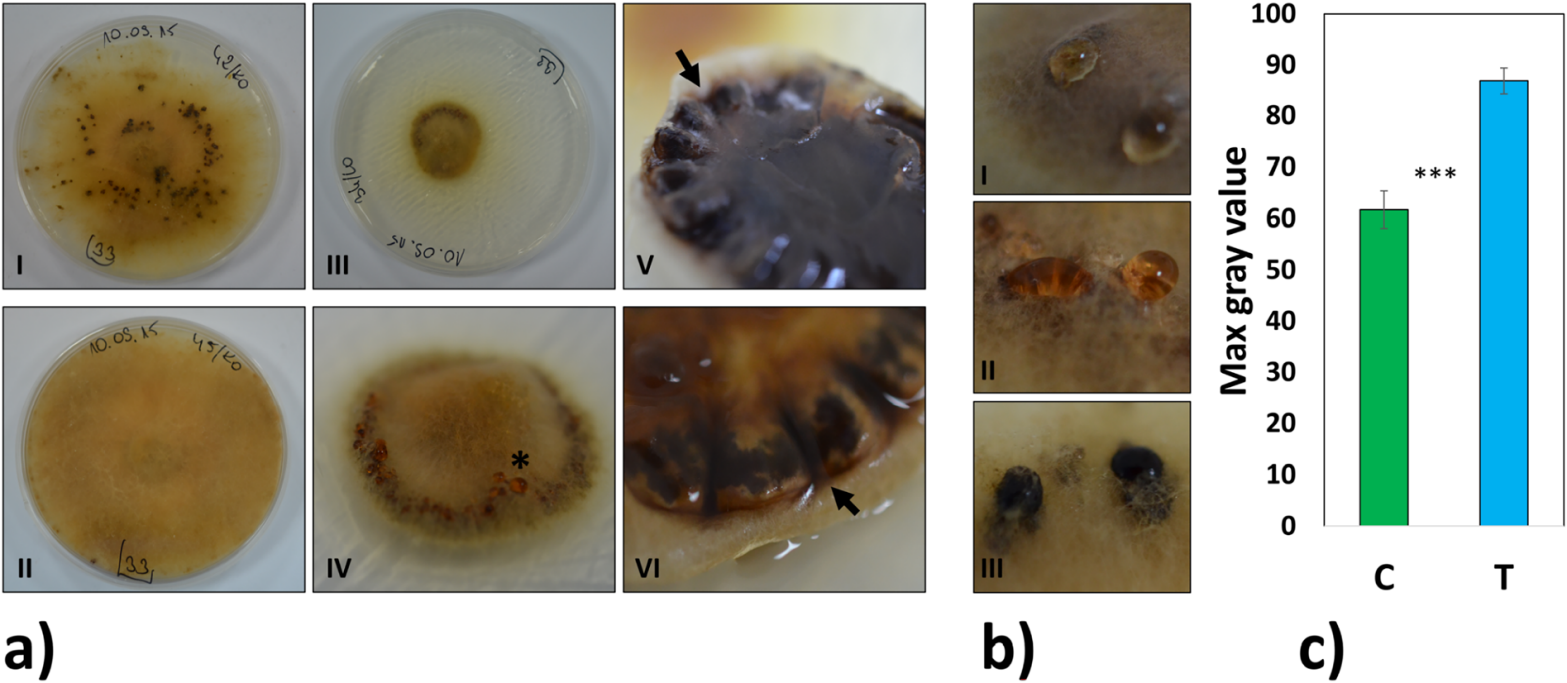
Treatment overview. Representative images, of analysed treatments (a). Panels I and II: Control mycelia (C). Panels III–VI: *P. involutus* maintained in low temperature (T), including images of mycelium bottom surfaces (Panels V and VI) with visible phenotypic changes, and bulges (arrows) responsible for multisclerotial‐like structures visible on panel IV. Mycelia exudates produced by sclerotial bodies and sclerotia‐like structures (b), differing in the intensity of their pigmentation (Panels I–III), see also the asterisk on panel IV. Close‐ups of mycelia represent the spectrum of exudate droplet staining observed in both experimental variants. Agar growth media pigmentation measured as the maximum grey value (an unitless pixel intensity value; c). Mean values ± SE are presented (*n* = 12). ***p < 0.001 according to the *t*‐test (*α* = 0.05). High‐quality images of mycelium were obtained during material harvesting, after 6 weeks of growth at a given temperature.

**FIGURE 2:**
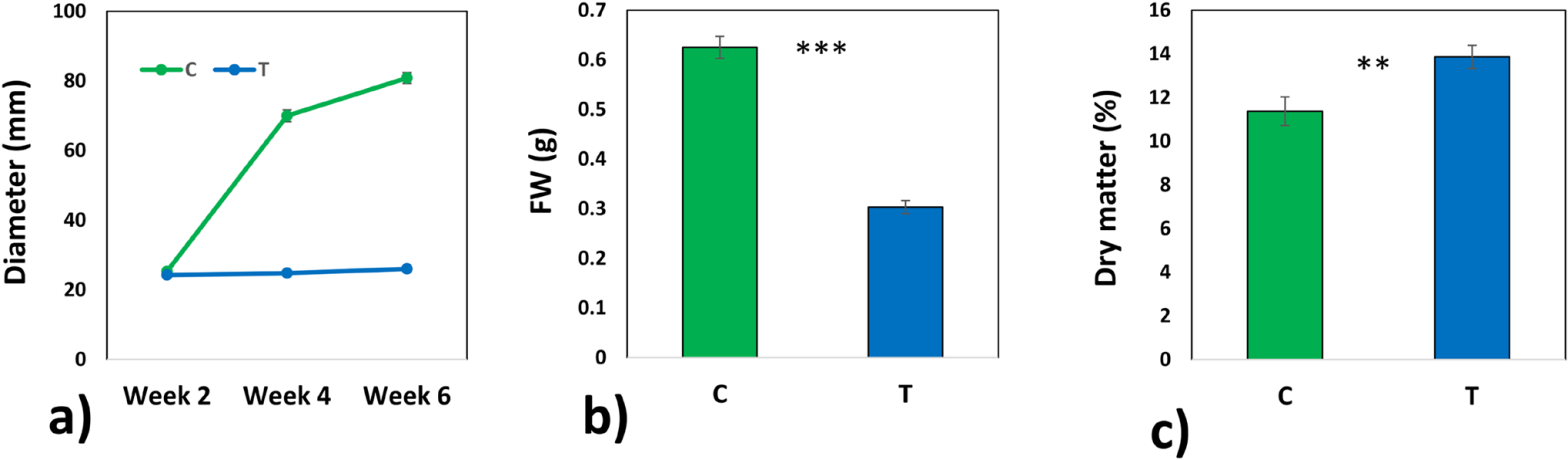
Biometric parameters of mycelia. Diameter (a), fresh mass (b) and dry matter percentage (c) of mycelia measured after 6 weeks of growth. Mean values ± SE are presented (*n* = 30). Significant differences according to the *t*‐test (*α* = 0.05). ***p < 0.001; ** 0.001 < *p* < 0.01.

**FIGURE 3:**
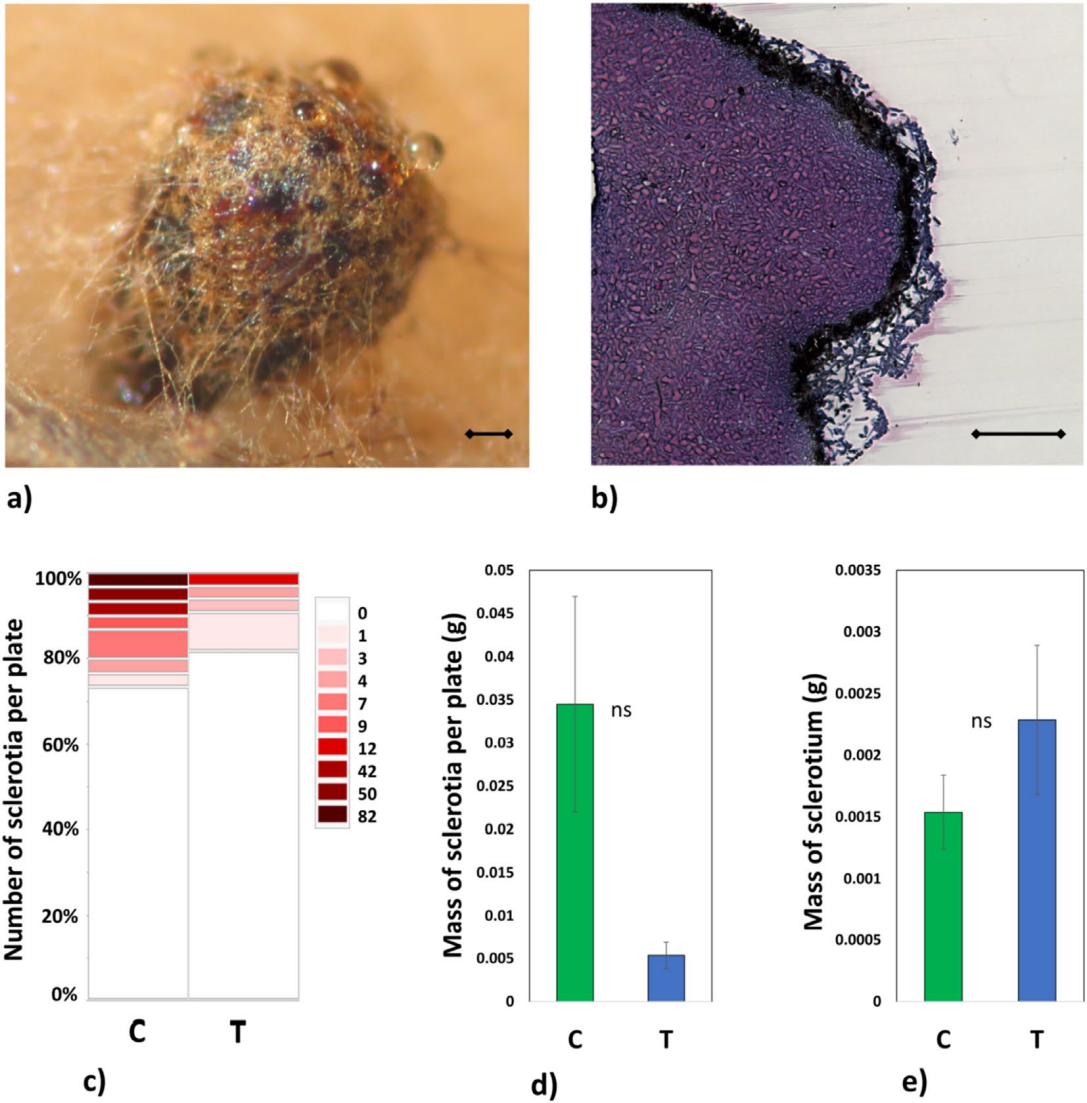
Sclerotial bodies. Representative sclerotium body (a) and its cross section (b; scale bar: 0.1 mm). Number of sclerotial bodies per plate presented as the mosaic plot showing the global distribution of the number (or lack thereof) of sclerotial bodies detected on individual plates. The particular panel size refers to the proportion of plates with a given number of sclerotia (expressed as a (%) of total number of analysed plates (*n* = 30) (c)); mass of all sclerotia detected on a particular plate (d), and mass of a single sclerotium (e). No significant differences were found between treatments (for d and e) according to the *t*‐tests (*α* = 0.05). ns—not significant.

Low temperature exposure caused a drastic decrease in the radial growth of *P. involutus* mycelia (Figures 1a and 2a). After 6 weeks of exposure to low temperature, the mycelium covered 28.9% ± 0.89% of the plate surface compared to 89.8% ± 1.74% coverage of the agar medium observed for mycelium growing under control conditions. Moreover, low temperature also triggered phenotypic changes, namely the thickening/folding of mycelia (Figure 1a panel IV, V and VI, arrows). This thickening/folding was frequently accompanied by sclerotia‐like structures on the surface of the mycelia (Figure 1a panel IV, asterisk), whereas classical sclerotial bodies were observed under both treatments (Figure 1a panel I or IX, Figure 3). In vitro cultivated *P. involutus* produced sclerotia with square structures composed of dense mycelia (Figure 3a,b). In this study, they tended to occur more frequently (on the higher number of Petri plates) in the mycelia cultivated in control conditions (*p* = 0.064; Figure 3c). Because the average masses of individual sclerotia were very similar in both treatments (Figure 3d), the total mass of sclerotia per plate also tended to be higher in the control treatment (*p* = 0.069; Figure 3e). Some of the *P. involutus* mycelia (regardless of the experimental variant), especially in the area around the sclerotial bodies and sclerotia‐like structures, produced drops of differently pigmented exudates (Figures 1b and 3a). The agar growth media for mycelia under low temperature conditions had significantly higher pigmentation compared to controls (Figure 1c), which was most probably caused by the same or similar exudates. The fresh and dry mass of mycelia were lower under the low temperature treatment compared to those under control conditions (Figure 2b and Appendix B). Low temperature also triggered a significant increase in dry matter percentage (Figure 2c).

### 3.2 Biochemical Features of Mycelia

Low temperature exposure did not change the carbon percentage in dry *P. involutus* mycelia (when normalised on FW, a 22.3% increase in C level in the low temperature variant was observed; Table 1). At the same time, low temperature caused a significantly higher increase in *N* (%) (which resulted in a decrease in the C/N ratio; Table 1). Moreover, the majority of analysed elements had lower abundances in mycelia exposed to low temperature relative to control (Table 1).

**FIGURE 4:**
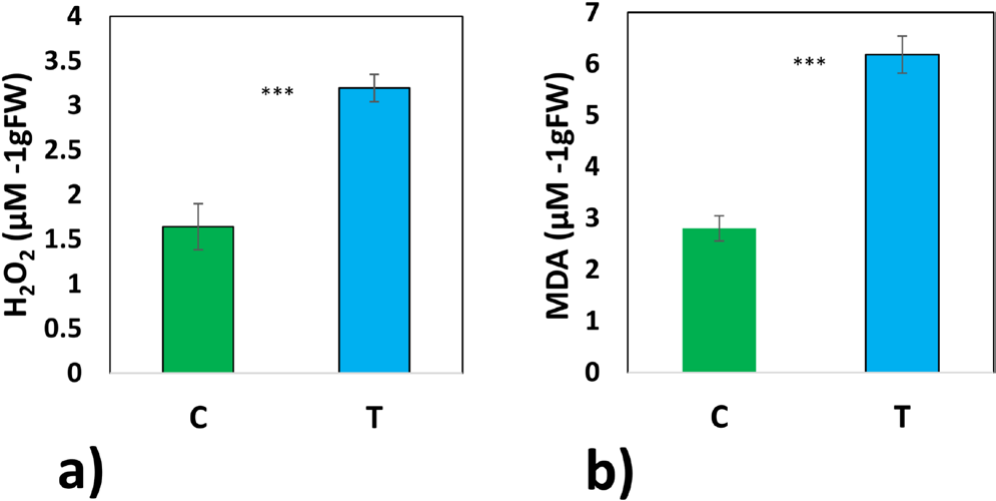
Biochemical features of mycelia. Concentration of H_2_O_2_ (a) and concentration of MDA (b), both analysed in *Paxillus involutus* mycelia after 6 weeks of growth. Data were normalised with FW. Mean values ± SE are presented (*n* = 6). Significant differences according to the *t*‐test (*α* = 0.05). ***p < 0.001.

H_2_O_2_ concentration in frozen mycelium was higher in mycelia exposed to low temperature, compared with control (Figure 4a). Lipid peroxidation levels estimated based on MDA concentrations were two‐fold higher under low temperature than in control (Figure 4b).

### 3.3 *P. involutus* Metabolome (GC MS/MS Data)

**FIGURE 5:**
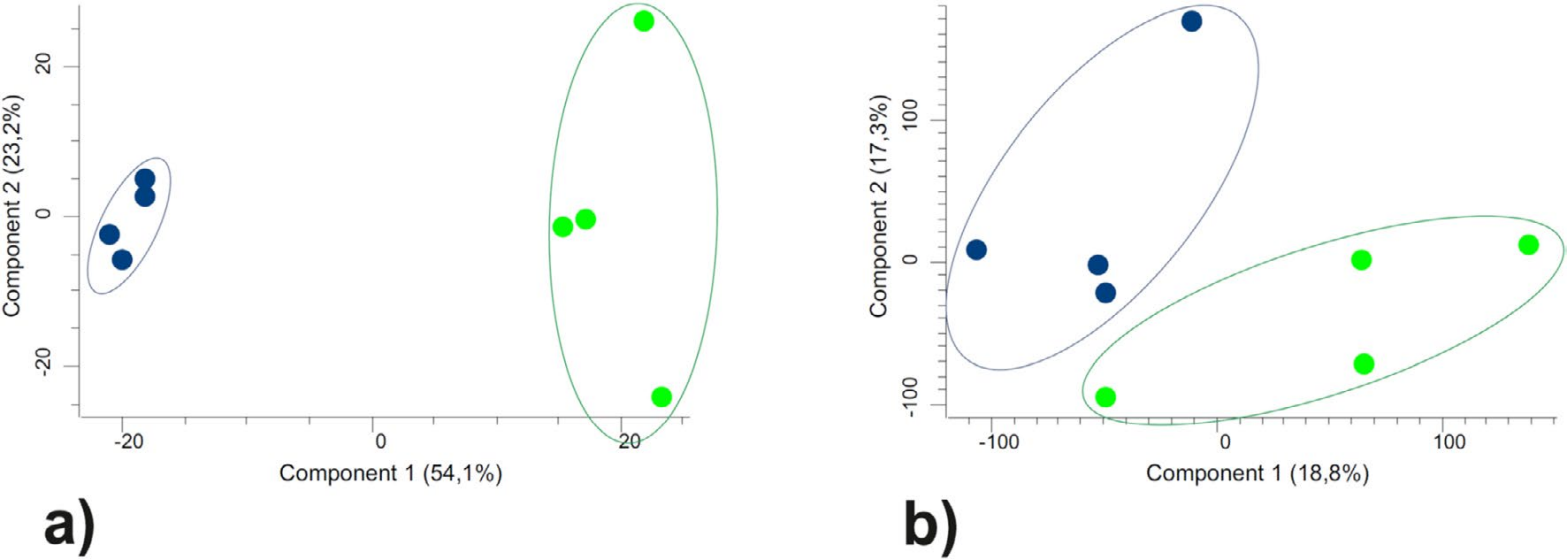
Principal component analysis of metabolite composition detected in mycelium extracts (*n* = 4). Score plots showing the variation in the determined metabolites (a), and the score plots showing the variation in the overall (identified into the molecular formula and unidentified) metabolites (b). Green points: control (C) treatment and dark blue points represent mycelia exposed to low temperature (T). Points representing biological repetitions of particular treatments are marked by the areas in the corresponding colours. Analysis was performed using the Perseus 1.6.15.0 software.

125 compounds were successfully identified in the mycelium (for a complete list of identified and unidentified compounds, see Appendix C). The most commonly detected compounds in the GC MS/MS study of *P. involutus* mycelia were O‐glycosides, followed by sugars mainly hexoses, sugar alcohols, various sugar acids and their derivatives, and amino acids (which altogether represented half of the detected compounds; Appendix C). About half of the identified molecular formula compounds were affected by the tested low temperature conditions (Appendix C). This significant impact of low temperature exposition on the mycelia metabolome was reflected in the results of PCA (Figure 5). Moreover, low temperature affected more significantly known/identified compounds (suggested a more significant effect on primary metabolism) as the distance between the analysed variants on the PCA graphs was bigger when compared to the identified compounds (Figure 5a) than to all detected ones (Figure 5b).

**FIGURE 6:**
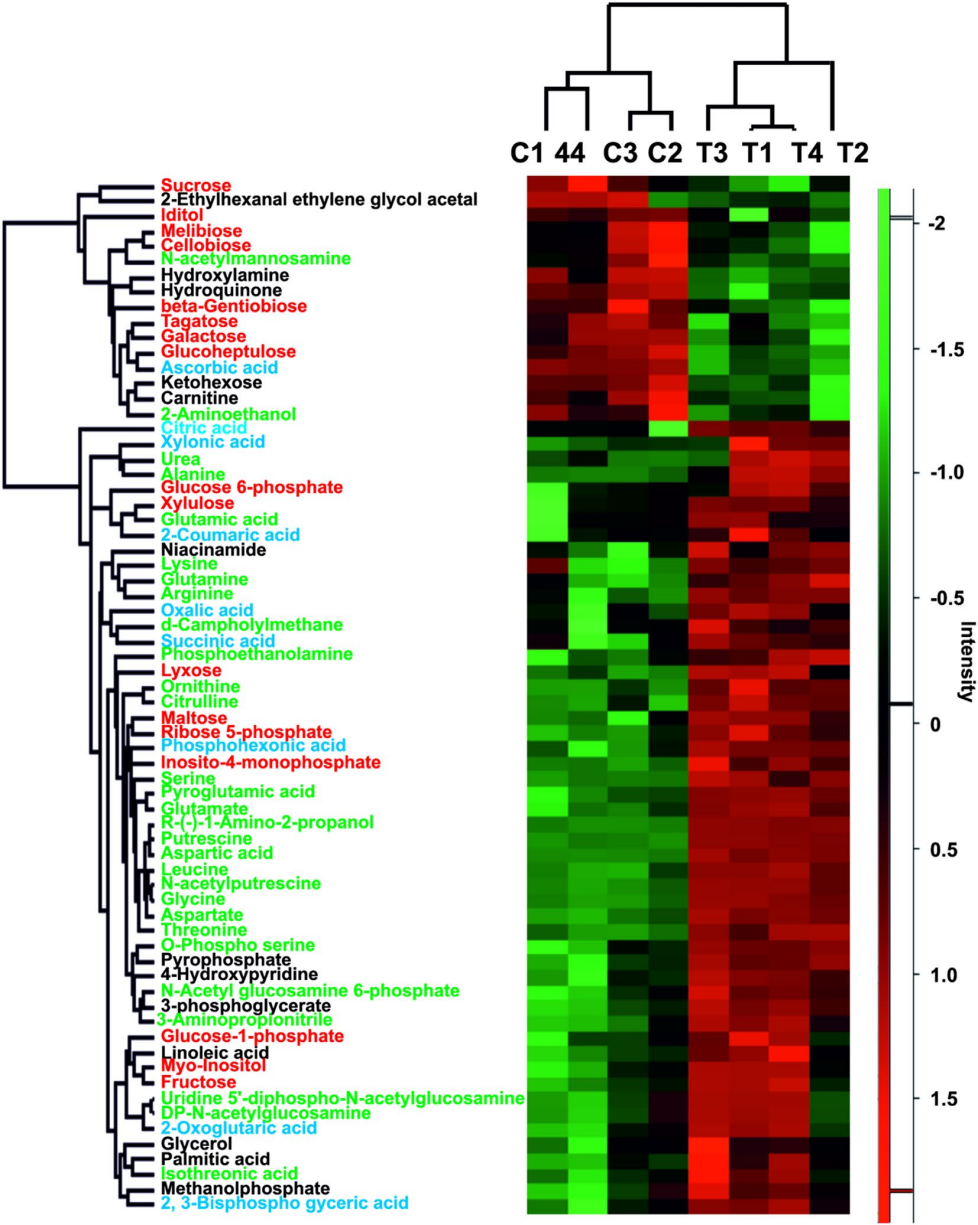
Heat map analysis combined with hierarchical cluster analysis showing determined metabolites that were differentially abundant between the analysed treatments (*p* < 0.05, *T*‐test). Intensity values were log 2‐transformed, batch‐corrected and *Z*‐scored rowwise. Green, minimal abundance; red, maximal abundance. Analysis was performed using the Perseus 1.6.15.0 software. Compound names colour code: Blue organic acids; green: Amino acids and other main N‐compounds; red: Carbohydrates (including phosphorylated sugars) and their alcohols; black remained compounds.

An overall of 52 particular compounds were more abundant and 16 less abundant under low temperature‐exposed mycelia compared to controls (Appendix C; Figure 6). The compounds with the largest decrease in abundance under low temperature were ascorbic acid, tagatose, glucoheptulose, and galactose, along with several other carbohydrates (sucrose, cellobiose and melibiose); (Appendix C and Figure 6). The metabolites with decreased abundance under low temperature treatment were involved mainly in starch, sucrose, and galactose metabolism (Appendix C). In the same time, trehalose (a major storage substance in mycelia and a well‐known fungal anti‐stress compound; (Ocón et al. 2007)) and inositol family members (compounds related to activating abiotic stress tolerance; (Jia et al. 2019)) were, or tended to be, more abundant in mycelia grown at 4°C (Appendix C).

Mycelia exposed to low temperature also had higher abundances of certain sugars (mainly maltose and fructose) and fatty acid, both saturated and polyunsaturated (palmitic and linoleic acid). They were also characterised by more glycolysis intermediates and organic acids important for central metabolism (citric and succinic acids) (Figure 6, Appendix C).

The amino‐2‐propanol (isopropanolamine) was the compound most increased in abundance under low temperature (Figure 6 and Appendix C), followed by putrescine, aspartic acid, and numerous other N‐compounds. The N‐compounds with the largest increase in abundance under low temperature were, for example, glycine, glutamic acid, glutamine, O‐phospho serine, ornithine, arginine, aspartate, alanine, and other amino acids (e.g., threonine). Only two N‐compounds showed reduced abundance in mycelia exposed to low temperature (N‐acetylmannosamine and 2‐aminoethanol) (Figure 6; for full GC MS/MS results, see Appendix C).

Metabolites enriched under low temperature are mainly involved in amino acid metabolism, but also in dicarboxylate and sucrose metabolism or citrate cycle (Appendix C).

Tryptophan and tyrosine were not detected while being regularly detected during our previous GC MS/MS experiments, (for example, Szuba, Marczak, Ratajczak, Kasprowicz‐Maluśki, et al. 2020), which indicates that their levels were very low in all analysed variants. Phenylalanine levels did not differ significantly between both treatments (Appendix C). Phenolic compounds were not significantly affected by temperature treatment with the exception of 2‐coumaric acid, which was increased under low temperature treatment (Appendix C).

Intermediates of the last steps of chitin polymer biosynthesis, namely N‐Acetyl glucosamine 6‐phosphate and UDP‐N‐acetylglucosamine were more abundant in the treated mycelia (Figure 6).

Moreover, despite that we did not identify directly N‐acetylglucosamine (GlnNAc), N‐acetylhexamine possibly being GlnNAc was detected in *P. involutus* mycelia (with abundance tending to be more abundant under low temperature treatment Appendix C).

**FIGURE 7:**
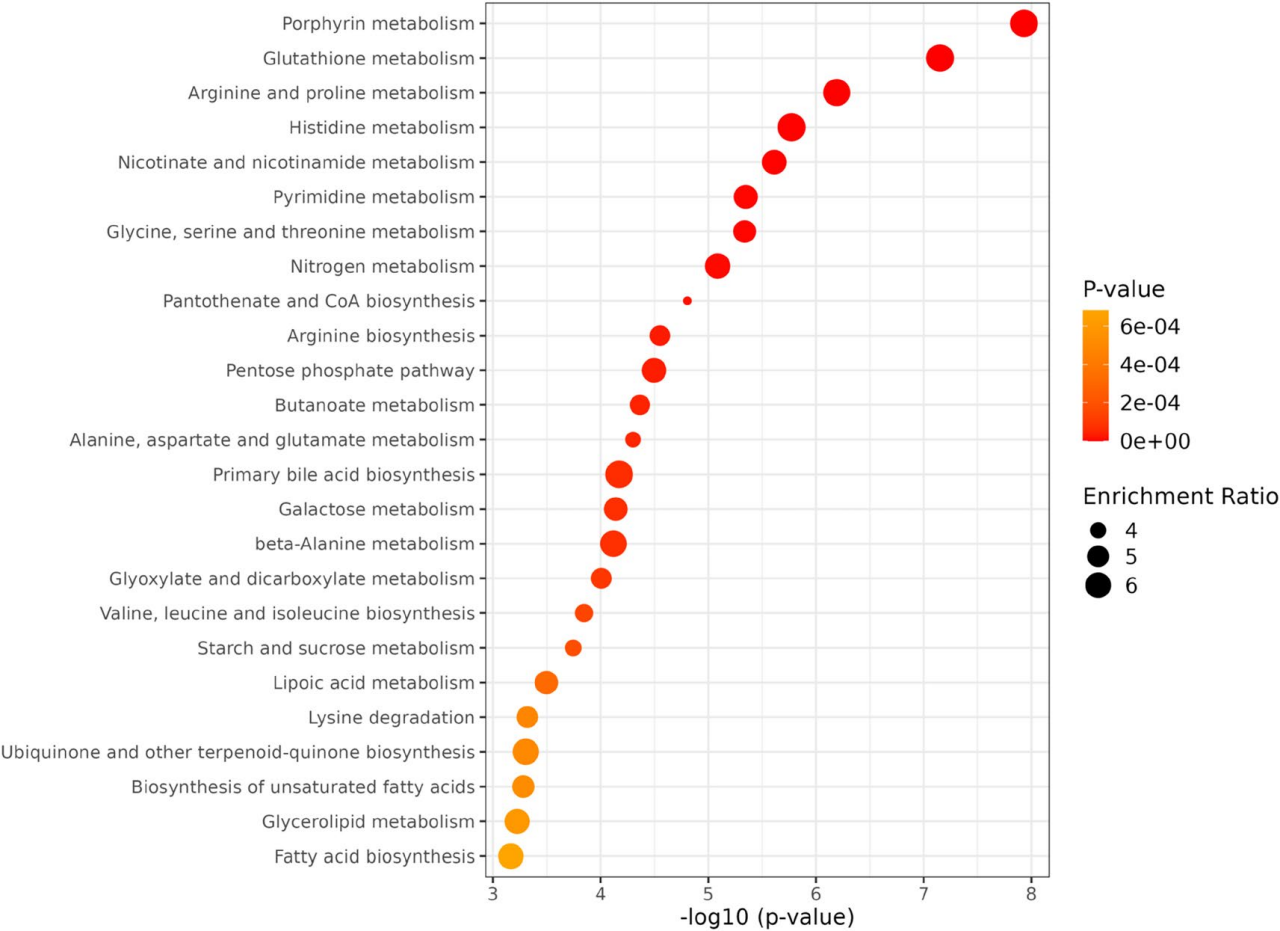
Quantitative enrichment analysis. The intensities of all identified compounds (for both treatments) were subjected to the analysis. The metabolic pathways are represented as circles according to their scores from the enrichment (vertical axis; −log(p)) and topology analyses (pathway impact, horizontal axis). Darker circle colours indicate more significant changes of metabolites in the corresponding pathway. The size of the circle corresponds to the pathway impact score. Analysis was performed using the MetaboAnalyst 6.0 (https://www.metaboanalyst.ca/) platform. Detailed information on the number of hits, *p* and FDR values: see Appendix C.

Among the molecular pathways most significantly affected by chronic low temperature exposition (according to the quantitative pathway enrichment analysis) were those involved in wide‐ranging carbohydrate and amino acid metabolism. Signals of re‐organisation of mycelia metabolism, namely modification of glutathione metabolism pathway, pentose phosphate pathway, pathways involved in glyoxylate and dicarboxylate as well as sucrose metabolism or biosynthesis of unsaturated fatty acids, were detected (Figure 7). The majority of the affected metabolomic pathways were directly involved in particular amino acids metabolism. Among them the most affected was the arginine and proline, histidine, glycine, serine and threonine metabolic pathway (Figure 7). Low temperature also affected the metabolism of alanine, aspartate, glutamate, valine, leucine and isoleucine (Figure 7 and Appendix C).

Summarising, almost all N‐compounds were characterised by increased abundance in mycelia exposed to low temperature, whereas changes in the abundance of various sugars and their derivatives were differentially altered under low temperature exposure.

## 4 Discussion

### 4.1 Molecular Background of Low Temperature Response in *P. involutus* Mycelia

Decreased growth, as observed in this study, is a common fungal response to suboptimal conditions (De Oliveira and Tibbett 2018), typically resulting from reduced cellular respiration (Atkin and Macherel 2009; Liu et al. 2015) and/or suppressed synthesis of structural components, with resources redirected towards defensive compounds such as osmoprotectants, antioxidants, or phenolics (Jacob et al. 2004; Gill and Tuteja 2010; Caretto et al. 2015). Our findings indicate a shift in C‐metabolism towards the production of known anti‐stress compounds like trehalose and inositol (Ocón et al. 2007; Jia et al. 2019).

**FIGURE 8:**
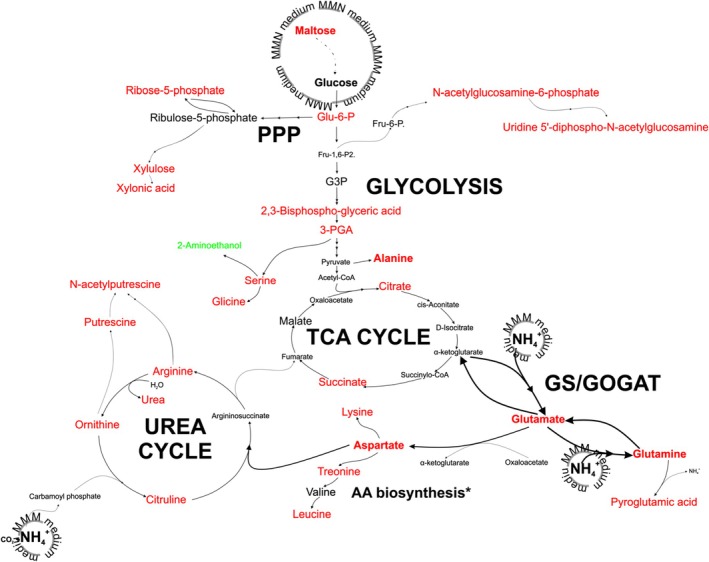
Summary‐ central metabolism modifications. A simplified diagram of the main N‐ and C‐ metabolism shifts observed in the central metabolism of *Paxillus involutus* mycelia grown under abiotic stress conditions. The main metabolic processes are marked in the figure (glycolysis, urea cycle, TCA cycle—the tricarboxylic acid cycle; PPP—the pentose phosphate pathway; GS/GOGAT—glutamine synthetase/glutamate synthase pathway). Red names: Compounds identified as more abundant in mycelia exposed to low temperature; green names—compounds found to be less abundant both in mycelia exposed to low temperature, both compared to controls (for details, see Appendix C). The thicker lines and metabolites highlighted in bold represent the presumed most important components of N uptake (suggested by increased N levels) in *P. involututs* mycelia grown under low temperature stress. The processes relating to presumed N uptake (reflected in observed increased N level) and C‐skeleton biosynthesis are marked in the figure. The N and C sources present in the MMN medium (maltose, glucose and NH_4_^+^) are marked by the circles. All *P. involutus* mycelia were grown in the same agar medium (MMN), supplemented with NH_4_^+^ as the sole N source (Kottke et al. 1987). Note that amino acid (AA) biosynthesis also occurred via intermediates of glycolysis.

In *P. involutus* mycelia exposed to low temperatures, we observed marked increases in late‐stage chitin biosynthesis substrates (Figure 8; (Merzendorfer 2011)), likely associated with cell wall remodelling. This coincided with increased cell dry weight (%) and thickened mycelial structure. While the impact of low temperatures on ECMF cell walls is not well understood, plant studies show cold acclimation enhances cell wall integrity and biosynthesis (Le Gall et al. 2015), suggesting that similar processes may occur in *Paxillus*.

Under low temperature, several key anti‐stress compounds showed no change or declined, including mannitol, a major fungal cryoprotectant (Tibbett et al. 2002), and ascorbic acid, a cellular antioxidant that is often undetectable in ECMF (including *P. involutus* ) and whose role in fungi remains unclear (Gill and Tuteja 2010; Ott et al. 2002). A notable decrease was observed in 2‐Aminoethanol, a major phospholipid head group and membrane disintegration product under stress (Rajaeian et al. 2011). While its release can enhance ROS detoxification and signal stress responses (Rajaeian et al. 2011), the inverse trend with MDA suggests that 2‐Aminoethanol was rather redirected towards phospholipid biosynthesis (e.g., phosphatidylethanolamine, phosphatidylcholine), potentially mitigating the effects of low temperature on membrane fluidity (Kwon et al. 2012; Chen et al. 2010).

Phenolic upregulation is a common stress response (Gill and Tuteja 2010; Caretto et al. 2015), yet the shikimate pathway was largely unchanged in this study. While aromatic L‐amino acids and related metabolites can serve as precursors for *Paxillus* pigments—for example, involutin derived from atromentin, synthesised from L‐tyrosine (Braesel et al. 2015)—L‐tyrosine was not detected in the present study. Both media variants showed dark pigmentation, especially under low temperature, a response linked to fungal cold adaptation (Hassan et al. 2016). Such pigmentation typically results from fungal pigments or oxidised polyphenols (Braesel et al. 2015; Shah et al. 2015), but no corresponding increase in these compounds was found, leaving the pigmentation source unclear and warranting further investigation.

The above‐mentioned defensive compounds are typically synthesised to counteract macromolecular damage from ROS (Alvarez et al. 2009; Sharma et al. 2012), which were elevated in this study. The lack of massive anti‐stress compound upregulation under low temperature may suggest either mild stress or partial acclimation due to prolonged exposure, rather than severe stress. These findings could also be related to mycelial dehydration, as indicated by increased dry matter content, which may reduce cold tolerance (Lehto et al. 2008). Overall, the limited stress response, together with metabolome‐inferred changes in cell wall and membrane function, suggests activation of cold tolerance mechanisms. This is consistent with the natural adaptation of ECMF, including *Paxillus*, to the temperate climates of the Northern Hemisphere (https://unite.ut.ee/bl_forw_sh.php?sh_name=SH1370017.10FU#fndtn‐panel1) (Szuba, Marczak, and Kozlowski 2020).

Finally, we found that *P. involutus* mycelia formed sclerotia, structures typically induced under suboptimal conditions, including low temperature (Moore and Peterson 1992; Cagigal and Sanchez 2017). While previous research reported smaller sclerotia at low temperatures (Moore et al. 1991), we observed similar sclerotial sizes across treatments, with greater abundance under control conditions. Thus, sclerotia formation does not appear to be specifically triggered by low temperature exposure.

### 4.2 Nitrogen Level Was Increased in *P. involutus* Mycelia Exposed to Low Temperature

In contrast to the typical reduction in cellular metabolism and respiration under abiotic stress (Pietikainen et al. 2005), *Paxillus* displayed increased N‐metabolism at 4°C (Figure 8), despite reduced mycelial growth and mild stress symptoms.

Nutrient acquisition and transfer to host plants are key ECMF functions (Szuba, Marczak, Ratajczak, Kasprowicz‐Maluśki, et al. 2020; Shantz et al. 2016), but are expected to decline at low temperatures due to reduced diffusion and enzyme activity (Tibbett and Cairney 2007). Indeed, numerous elements had lower concentrations in *Paxillus* mycelia exposed to low temperature, confirming such an assumption.

At the same time, the glutamine synthetase—glutamate synthase (GS—GOGAT) pathway was significantly upregulated under low temperature (Figure 8), consistent with its known role in fungal N assimilation via glutamine and glutamate biosynthesis (Martin et al. 1994; Chalot et al. 1994; Walker and Van Der Donk 2016). In ECMF utilising NH_4_^+^, glutamate transamination typically leads to alanine and arginine accumulation (Martin et al. 1994; Chalot et al. 1994), as observed in this study. This suggests enhanced NH_4_^+^ uptake and metabolism, with increased synthesis of glycolysis‐derived C‐skeletons for N‐compound production—despite no corresponding biomass increase (Szuba, Marczak, Ratajczak, Kasprowicz‐Maluśki, et al. 2020) (Figure 8).

Although low temperature can reduce C flux in symbiosis (Usman et al. 2021), previous studies showed that C and N acquisition in ECMF are not strictly coupled (Bogar et al. 2022; Szuba, Marczak, Ratajczak, Kasprowicz‐Maluśki, et al. 2020). This is consistent with our findings, which revealed elevated N levels under low temperature despite stable C content and redirected C metabolism. Given that ECMF rely on host‐derived C under natural conditions (Szuba, Marczak, Ratajczak, Kasprowicz‐Maluśki, et al. 2020), this decoupling of C and N metabolism observed in free‐living mycelia may also have ecological significance during cold periods, when plant C supply is limited, for fungi living in symbiosis with trees.

Despite cold temperatures negatively affecting mycelial function, their impact on fungal N levels is generally limited (Zhang et al. 1995; Karasawa et al. 2012), though the underlying mechanisms remain unclear. One hypothesis involves cold‐active extracellular enzymes enhancing organic N uptake (Tibbett, Sanders, Minto, et al. 1998; Tibbett, Sanders, and Cairney 1998), which could help counteract the limited availability of inorganic N in the soil pool in nature (Tibbett and Cairney 2007). Nevertheless, in the present study, *Paxillus* grew solely on inorganic N media (Szuba, Marczak, Ratajczak, Kasprowicz‐Maluśki, et al. 2020; France and Reid 1984). Therefore, the observed increase in N metabolism under low temperature suggests that this effect was not due to enhanced organic N uptake (Kottke et al. 1987).

While high temperatures generally enhance inorganic N uptake (Lilleskov et al. 2011; Solly et al. 2017), lower temperatures are often thought to reduce nutrient availability—supported here by decreased nutrient concentrations in cold‐exposed mycelia. However, in this study, *P. involutus* , a nitrophilic ECMF, likely exhibited increased inorganic N uptake at low temperature. Similarly, in ectomycorrhizal *Eucalyptus*, only NH_4_^+^ uptake increased under cold conditions (Warren 2009). This may explain the elevated N content and enhanced N metabolism in *Paxillus* grown in NH_4_^+^‐based MMN medium under low temperature (Kottke et al. 1987).

GlcNAc is involved in cellular regulation, nutrient sensing, and may influence fungal virulence pathways (Min et al. 2020). We speculate that changes in GlcNAc‐related metabolites in low temperature‐exposed mycelia could affect not only cell wall structure and N levels, but also possible host–fungi interactions in ECM. Such shifts may contribute to the disruption of mutualism in *Paxillus* under climate change (Szuba, Marczak, Ratajczak, Kasprowicz‐Maluśki, et al. 2020; Rudawska and Leski 2019), though further research is needed to confirm this hypothesis.

Mycelia in this study were exposed to 4°C for 6 weeks, so their molecular status is likely to be more similar to acclimatised or adapted than to stressed fungi. Cold adaptation in psychrophilic fungi involves increased unsaturated fatty acid biosynthesis for membrane fluidity and cryoprotectant accumulation, such as glycerol (Su et al. 2016). Similarly, we observed elevated levels of a major polyunsaturated fatty acid in *P. involutus* at 4°C. Studies on psychrophilic fungi exposed to 4°C also reported increased protein synthesis and upregulation of Gly and Arg codons under low temperature (Su et al. 2016), consistent with our findings of higher N%, enhanced N metabolism, and elevated Gly and Arg (Figure 8). These results indicate that adaptation/acclimatisation mechanisms may involve not only improved N acquisition (Solly et al. 2017; Tibbett and Cairney 2007; Tibbett, Sanders, Minto, et al. 1998) but also early activation of inorganic N metabolism pathways.

Overall, our results show that low temperature led to increased N metabolism in mycelia, accompanied by a shift in C metabolism. This rather unexpected response suggests that N levels—and possibly uptake—may also rise in natural conditions during cooler periods, such as (leafless) spring and autumn. However, this hypothesis requires further environmental validation.

## 5 Conclusion

Storing mycelia for 6 weeks at 4°C caused an increase in oxidative stress indicators, accompanied by a decrease in several anti‐stress compounds. We also observed elevated levels of numerous N‐compounds. Meanwhile, low temperature exposure had variable effects on carbohydrate abundance, along with increased levels of glycolysis intermediates and inositol family members, while C (%) remained unchanged. This suggests that C‐metabolism was not reduced but rather redirected, most likely to provide C‐skeletons for enhanced biosynthesis of N‐compounds, including amino acids and metabolites associated with low temperature tolerance. Altogether, the changes in N‐compound abundance, accompanied by a significant increase in *N* (%) in fungal cells compared to the control, suggest a strong intensification of N‐metabolism under low temperature conditions.

As shown in our study, low temperature exposure may trigger mild stress symptoms in *P. involutus* strains cultivated in vitro and directly affect fungal metabolism. The observed increases in the N level should be interpreted in the context of inhibited mycelial growth. Nevertheless, the molecular response to low temperature exposure in vitro likely reflects the natural adaptation of ECMF to cold habitats, which constitute their typical environment.

## Author Contributions

**A.S.:** conceptualisation. **A.S. and Ł.M.:** methodology. **A.S. and Ł.M.:** software. **A.S. and Ł.M.:** validation. **A.S.:** formal analysis. **A.S. and A.S.:** investigation. **A.S. and Ł.M.:** resources. **A.S. and Ł.M.:** data curation. **A.S.:** writing – original draft preparation. **A.S., W.B.Ż., J.M., and Ł.M.:** writing – review and editing. **A.S., W.B.Ż., and J.M.:** visualisation. **A.S.:** supervision. **A.S.:** project administration. **A.S.:** funding acquisition. All authors have read and agreed to the published version of the manuscript.

## Conflicts of Interest

The authors declare no conflicts of interest.

## Supporting information


**Appendix A.** Detailed information on agar growth medium composition.

**Appendix B.** Biometrical and biochemical data normalised to the DW.

**Appendix C.** Metabolomic results: expanded data.

## Data Availability

The data that support the findings of this study are available on request from the corresponding author. The data are not publicly available due to privacy or ethical restrictions.
