# Supplementary material for: Low Temperature Enhances N‐Metabolism in Paxillus involutus Mycelia In Vitro: Evidence From an Untargeted Metabolomic Study
Source: Environ Microbiol. 2025 Aug 12;27(8):e70162. doi: 10.1111/1462-2920.70162 (PMC12343193; doi:10.1111/1462-2920.70162)
Supplement: Supplementary file 1 — Appendix A. Detailed information on agar growth medium composition. [file EMI-27-e70162-s001.docx]

**Low Temperature Enhances N‐Metabolism in *Paxillus involutus* Mycelia In Vitro: Evidence From an Untargeted Metabolomic Study**

Agnieszka Szuba, Weronika B. Żukowska, Joanna Mucha, Aleksander Strugała and Łukasz Marczak

**Appendix A**

**Detailed information on agar growth medium composition**

An agar medium was used for both the control (C) and low-temperature exposed (T) treatments.

A full MMN (detailed composition is given below) with an approximately 5 mm layer medium was used for experiments.

**MMN medium composition:**

| **Compound** | **Concentration (mg/L)** |
| --- | --- |
| Ammonium L-tartrate | 500 |
| KH2PO4 | 500 |
| MgSO4·7H2O | 500 |
| CaCl2·6H2O | 5 |
| MnSO4·5H2O | 5 |
| ZnSO4·7H2O | 0.025 |
| CuSO4·5H2O | 0.025 |
| Iron(III) citrate | 0.012 |
| Thiamine | 0.05 |
| Biotin | 0.005 |
| Glucose | 10 000 |
| Maltose | 5 000 |

Agar (BioShop; AGR002.1): 0.9 %. The final pH=5.6
