# Supplementary material for: Low Temperature Enhances N‐Metabolism in Paxillus involutus Mycelia In Vitro: Evidence From an Untargeted Metabolomic Study
Source: Environ Microbiol. 2025 Aug 12;27(8):e70162. doi: 10.1111/1462-2920.70162 (PMC12343193; doi:10.1111/1462-2920.70162)
Supplement: Supplementary file 2 — Appendix B. Biometrical and biochemical data normalised to the DW. [file EMI-27-e70162-s002.pptx]

## Slide 1
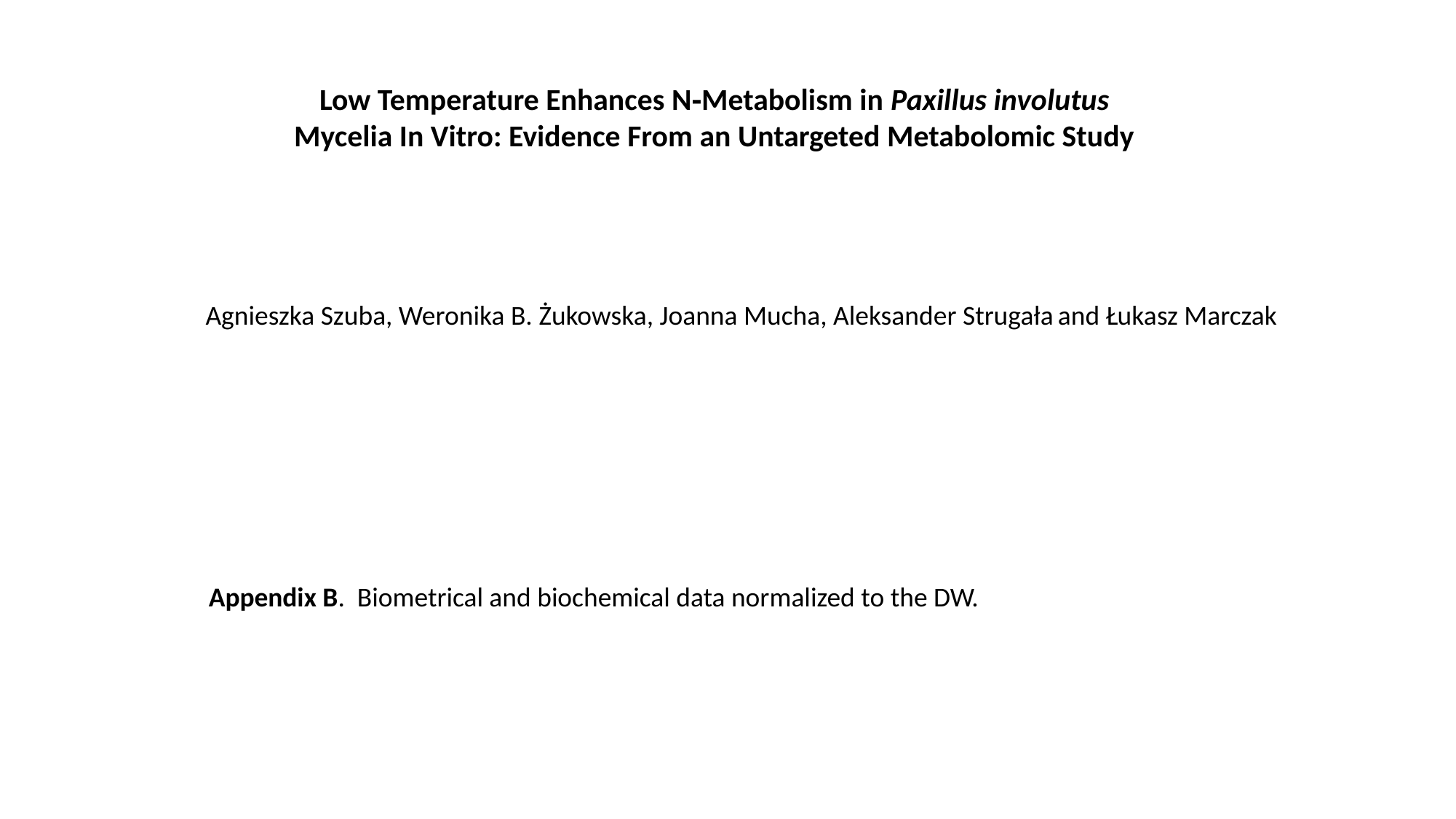

Low Temperature Enhances N‐Metabolism in Paxillus involutus
Mycelia In Vitro: Evidence From an Untargeted Metabolomic Study
Agnieszka Szuba, Weronika B. Żukowska, Joanna Mucha, Aleksander Strugała and Łukasz Marczak
Appendix B. Biometrical and biochemical data normalized to the DW.

## Slide 2
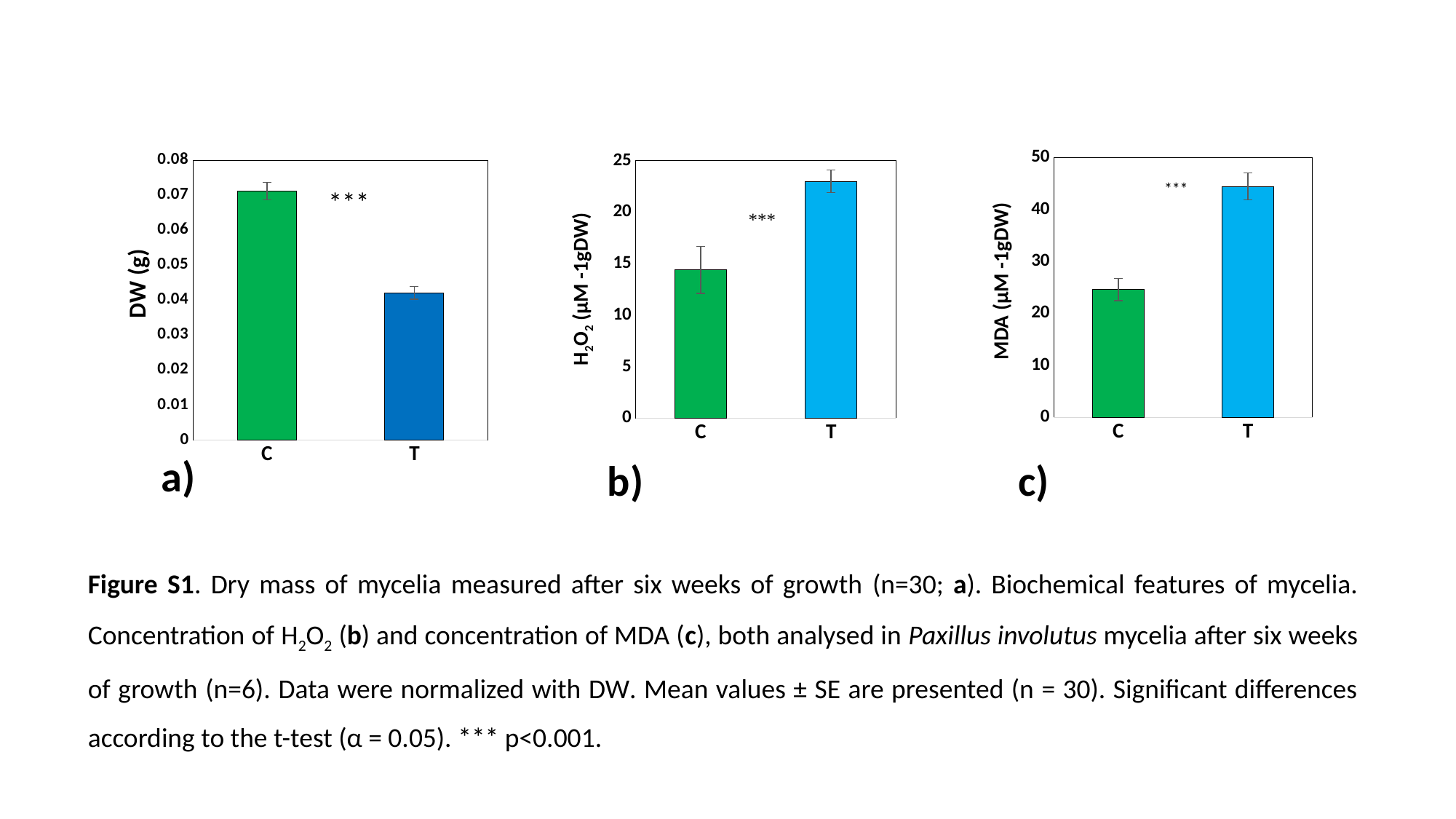

### Chart
| Category | |
|---|---|
| C | 24.624348 |
| T | 44.489034 |
### Chart
| Category | |
|---|---|
| C | 14.418574 |
| T | 23.001145 |
### Chart
| Category | |
|---|---|
| C | 0.07116 |
| T | 0.042111 |***
MDA (μM -1gDW)
DW (g)
H2O2 (μM -1gDW)
a)
b)
c)
Figure S1. Dry mass of mycelia measured after six weeks of growth (n=30; a). Biochemical features of mycelia. Concentration of H2O2 (b) and concentration of MDA (c), both analysed in Paxillus involutus mycelia after six weeks of growth (n=6). Data were normalized with DW. Mean values ± SE are presented (n = 30). Significant differences according to the t-test (α = 0.05). *** p<0.001.

## Slide 3
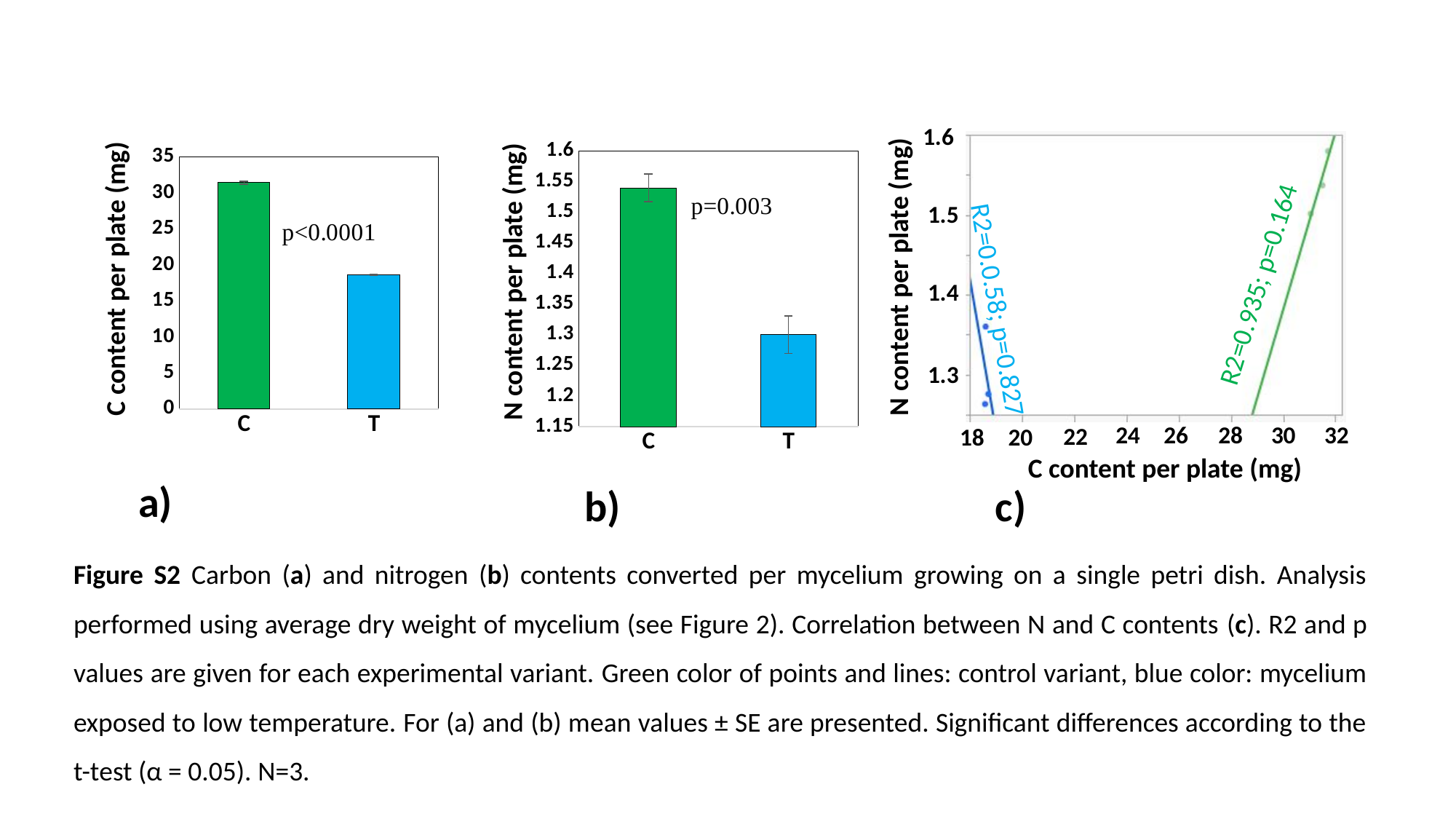

1.6
### Chart
| Category | C content |
|---|---|
| C | 31.428868 |
| T | 18.64118 |
### Chart
| Category | n content |
|---|---|
| C | 1.5394215 |
| T | 1.2998293 |1.5
C content per plate (mg)
N content per plate (mg)
N content per plate (mg)
R2=0.935; p=0.164
1.4
R2=0.0.58; p=0.827
1.3
24
26
32
30
28
22
18
20
C content per plate (mg)
a)
b)
c)
Figure S2 Carbon (a) and nitrogen (b) contents converted per mycelium growing on a single petri dish. Analysis performed using average dry weight of mycelium (see Figure 2). Correlation between N and C contents (c). R2 and p values are given for each experimental variant. Green color of points and lines: control variant, blue color: mycelium exposed to low temperature. For (a) and (b) mean values ± SE are presented. Significant differences according to the t-test (α = 0.05). N=3.
